# Supplementary material for: Modeling glioblastoma heterogeneity as a dynamic network of cell states
Source: Mol Syst Biol. 2021 Sep 16;17(9):e10105. doi: 10.15252/msb.202010105 (PMC8444284; doi:10.15252/msb.202010105)
Supplement: Supplementary file 5 — Source Data for Figure 3 [file MSB-17-e10105-s001.zip › Figure3A_sourcedata/GSEA_3065/nosigdb_state2.GseaPreranked.1622718419215/RICHARDS_LIDDELOW_NATURE_2017_A2_REACTIVE_ASTROCYTES.html]

Details for gene set RICHARDS\_LIDDELOW\_NATURE\_2017\_A2\_REACTIVE\_ASTROCYTES[GSEA]

|  || Dataset | state2 |
| Phenotype | NoPhenotypeAvailable |
| Upregulated in class | na\_neg |
| GeneSet | RICHARDS\_LIDDELOW\_NATURE\_2017\_A2\_REACTIVE\_ASTROCYTES |
| Enrichment Score (ES) | -0.7746936 |
| Normalized Enrichment Score (NES) | -2.3459494 |
| Nominal p-value | 0.0 |
| FDR q-value | 0.0 |
| FWER p-Value | 0.0 |
Table: GSEA Results Summary

  

Fig 1: Enrichment plot: RICHARDS\_LIDDELOW\_NATURE\_2017\_A2\_REACTIVE\_ASTROCYTES      
 Profile of the Running ES Score & Positions of GeneSet Members on the Rank Ordered List

  

| PROBE | GENE SYMBOL | GENE\_TITLE | RANK IN GENE LIST | RANK METRIC SCORE | RUNNING ES | CORE ENRICHMENT || 1 | TUBB6 |  |  | 622 | 0.160 | -0.0277 | No |
| 2 | S100A10 |  |  | 2608 | 0.044 | -0.2280 | No |
| 3 | IFI16 |  |  | 3054 | 0.033 | -0.2674 | No |
| 4 | HSPB1 |  |  | 3629 | 0.022 | -0.3232 | No |
| 5 | HMGA1 |  |  | 4135 | 0.013 | -0.3736 | No |
| 6 | B3GNT5 |  |  | 5120 | -0.002 | -0.4777 | No |
| 7 | S1PR3 |  |  | 5237 | -0.004 | -0.4891 | No |
| 8 | ASNS |  |  | 5539 | -0.009 | -0.5189 | No |
| 9 | GAP43 |  |  | 5863 | -0.014 | -0.5499 | No |
| 10 | SBNO2 |  |  | 6107 | -0.018 | -0.5713 | No |
| 11 | AKAP12 |  |  | 6471 | -0.025 | -0.6039 | No |
| 12 | OSMR |  |  | 7864 | -0.062 | -0.7370 | No |
| 13 | TNFRSF12A |  |  | 7931 | -0.064 | -0.7286 | No |
| 14 | VGF |  |  | 8046 | -0.069 | -0.7242 | No |
| 15 | TM4SF1 |  |  | 8150 | -0.074 | -0.7173 | No |
| 16 | VIM |  |  | 8691 | -0.110 | -0.7483 | Yes |
| 17 | ANXA2 |  |  | 8891 | -0.134 | -0.7373 | Yes |
| 18 | HMOX1 |  |  | 9084 | -0.163 | -0.7187 | Yes |
| 19 | CD109 |  |  | 9093 | -0.165 | -0.6801 | Yes |
| 20 | EMP1 |  |  | 9156 | -0.179 | -0.6438 | Yes |
| 21 | PTX3 |  |  | 9249 | -0.212 | -0.6028 | Yes |
| 22 | LGALS3 |  |  | 9308 | -0.236 | -0.5524 | Yes |
| 23 | GDF15 |  |  | 9322 | -0.247 | -0.4945 | Yes |
| 24 | TIMP1 |  |  | 9387 | -0.334 | -0.4212 | Yes |
| 25 | CTGF |  |  | 9400 | -0.375 | -0.3325 | Yes |
| 26 | CD44 |  |  | 9407 | -0.397 | -0.2380 | Yes |
| 27 | IGFBP3 |  |  | 9424 | -0.502 | -0.1194 | Yes |
| 28 | GFAP |  |  | 9425 | -0.504 | 0.0016 | Yes |
Table: GSEA details [plain text format]

  

Fig 2: RICHARDS\_LIDDELOW\_NATURE\_2017\_A2\_REACTIVE\_ASTROCYTES: Random ES distribution      
 Gene set null distribution of ES for **RICHARDS\_LIDDELOW\_NATURE\_2017\_A2\_REACTIVE\_ASTROCYTES**
